# Multidimensional QoE of Multiview Video and Selectable Audio IP Transmission

**DOI:** 10.1155/2015/417290

**Published:** 2015-05-27

**Authors:** Toshiro Nunome, Takuya Ishida

**Affiliations:** Department of Computer Science and Engineering, Graduate School of Engineering, Nagoya Institute of Technology, Nagoya 466-8555, Japan

## Abstract

We evaluate QoE of multiview video and selectable audio (MVV-SA), in which users can switch not only video but also audio according to a viewpoint change request, transmitted over IP networks by a subjective experiment. The evaluation is performed by the semantic differential (SD) method with 13 adjective pairs. In the subjective experiment, we ask assessors to evaluate 40 stimuli which consist of two kinds of UDP load traffic, two kinds of fixed additional delay, five kinds of playout buffering time, and selectable or unselectable audio (i.e., MVV-SA or the previous MVV-A). As a result, MVV-SA gives higher presence to the user than MVV-A and then enhances QoE. In addition, we employ factor analysis for subjective assessment results to clarify the component factors of QoE. We then find that three major factors affect QoE in MVV-SA.

## 1. Introduction

Multimedia services over IP networks have been very popular. In the services, high definition audiovisual media can be handled recently; users can feel a sense of really being there with the services. In this paper, we refer to the sense as “presence.”

The high definition audiovisual media are not the only way to provide the presence to the users. For giving higher presence to the users, MVV (*multiview video*), in which the users can change the viewpoint, has been studied [1].

The ultimate goal of the network services is to provide high QoE (*quality of experience*), which represents the overall acceptability of an application or service, as perceived subjectively by the end-users [2]. In order to enhance QoE, it is important to monitor the QoE perceived by the users and manage the network services on the basis of the QoE.

There have been many studies regarding MVV systems. However, most of the studies focus on the coding techniques such as MVC (*multiview video coding*) [3] and assess the effectiveness with the throughput and PSNR (*peak signal to noise ratio*), which measures spatial quality of video. Thus, few studies assess the effect of network delay and its jitter, which is the major characteristic in IP transmission, on QoE. In addition, those studies rarely consider audio; in real applications, audio and MVV are transmitted together.

In [4], for improving QoE of current video conferencing services, a real-time multicamera control and selection mechanism for the best view of human faces in Skype video conferencing is proposed. This paper mainly focuses on viewpoint selection algorithms, and the effect of delay and jitter in IP networks is not considered.

Apostu et al. have investigated the design of an intuitive user interface for MVV that makes effective use of available information about the spatial relationship of the individual perspectives [5]. This paper compares four user interfaces in terms of multidimensional QoE. They mainly deal with content creation and user interface design; then, the assessment does not include audio quality. In addition, the effect of IP transmission is not clearly considered.

On the other hand, Savas et al. have assessed the effect of channel capacity and packet loss in IP networks on QoE of MVV [6]. They employ a streaming mechanism based on interleaving streams of adjacent views and using unequal error protection. However, they do not consider audio and the effect of viewpoint change function in the assessment. In addition, they employ a scalar QoE measure, that is, not multidimensional.

In [7, 8], MVV-A (MVV* and audio*), which is MVV accompanied by audio, is transmitted over the IP network, and QoE assessment has been conducted. In the study, the users can select video by a viewpoint change request, but audio does not change according to the request. Requirements for multimedia services are not only beautiful video or audio but also feeling the sense of being there, that is, the presence. The selectable audio can give the users the presence and then can enhance QoE.

In this paper, we improve the MVV-A system in [7, 8] as the users can select not only video but also audio by the viewpoint change request. We refer to the improved system as MVV-SA (MVV* with selectable audio*). We compare MVV-SA with MVV-A from a QoE point of view.

In MVV-SA, the users can watch and listen audiovisual media of arbitrary viewpoints. Therefore, many factors such as quality of video and audio, viewpoint change response, and the presence affect QoE. Therefore, in this paper, we employ the semantic differential method [9], which evaluates QoE with many adjective pairs.

Moreover, in order to absorb network delay jitter, playout buffering control is carried out. In the MVV-SA system, the playout buffering control brings a tradeoff relation between viewpoint change response and audiovisual quality; it is a factor which affects QoE. Thus, this paper also assesses the effect of playout buffering time on QoE.

The remainder of this paper is organized as follows.  Section 2 introduces the MVV-SA system.  Section 3 describes the experimental method.  Section 4 presents experimental results.  Section 5 concludes this paper.

## 2. MVV-SA System

**Figure 1:**
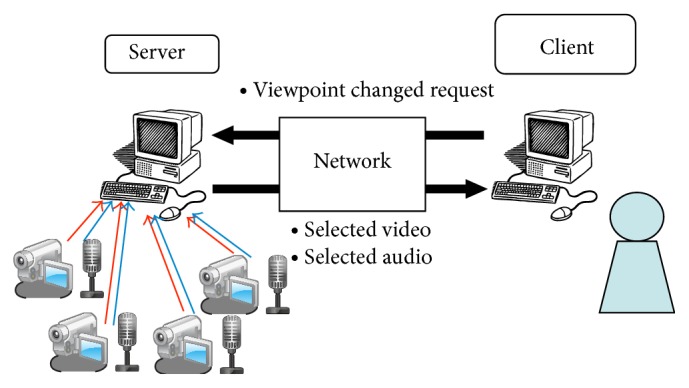
MVV-SA system.

MVV-SA is a system in which the user can watch and listen the audiovisual contents from various viewpoints while he/she chooses the viewpoints arbitrarily. It provides high flexibility of the service for the user.  Figure 1 shows a system model of the MVV-SA in this paper. The server provides the MVV-SA service to the client, and the user receives the service at the client.

In the MVV-SA system, multiple cameras are connected to the server; in this study, we consider four cameras. The server captures videos from the four cameras simultaneously and encodes them into separate video streams. It transmits one of the four video streams to the client according to the user's choice.

In the previous system named MVV-A, there is only one microphone connected to the server; the user cannot change audio because audio is captured at one place only. This paper enhances MVV-A to capture many audio streams. In Figure 1, audio is inputted from each video camera. Thereby, the user can also select audio with video.

**Figure 2:**
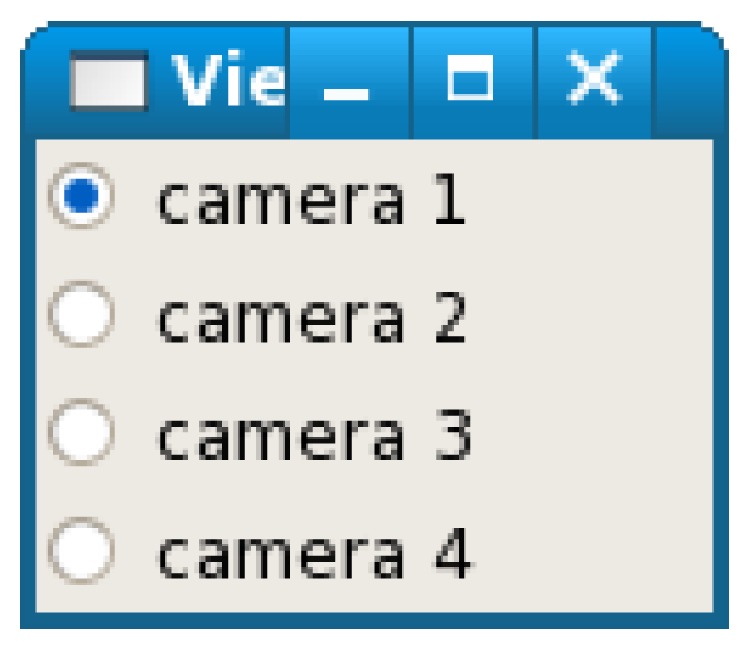
Viewpoint change GUI.

A GUI (graphical user interface) used for viewpoint change is shown in Figure 2; it appears on client display as a window of the X-window system. The user can change the viewpoint by clicking each radio button of the GUI. When the user clicks the button, the client transmits a viewpoint change request to the server for notifying the user's choice.

One of the merits of MVV-SA is provision of higher presence than MVV-A because the user can watch video and listen to audio on the camera's position. Meanwhile, there is some additional cost for capturing plural audio streams simultaneously in MVV-SA, for example, capture devices, interfaces, and computational cost. This paper assesses the fundamental effect of the selectable audio on QoE, and then the tradeoff between the cost and the enhancement of QoE is considered as a future study issue.

## 3. Experiment

### 3.1. Experimental System

**Figure 3:**
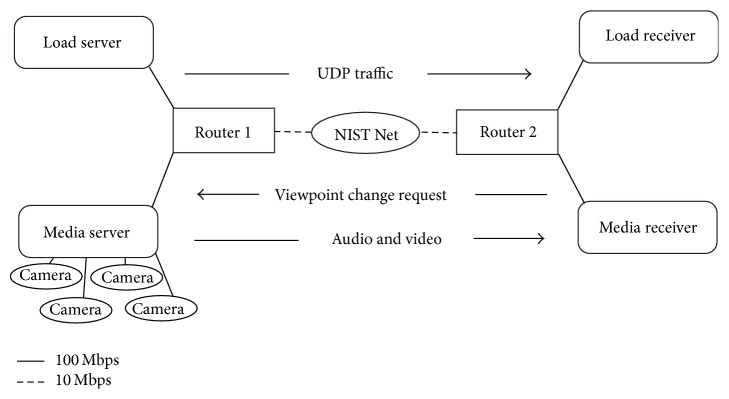
System configuration.

Figure 3 shows the configuration of the experimental system. Media server is the server of MVV-SA, and media receiver is the client of MVV-SA. Load server is the server of the load traffic, and load receiver is the client of the load traffic. NIST Net, which is a PC, is laid out between the routers. Both Router 1 and Router 2 are Riverstone's RS3000. Router 1 and Nist Net, and Router 2 and Nist Net are connected by a full duplex Ethernet line of 10 Mbps. All the other links are 100 Mbps Ethernet.

Four SONY HDR-CX180 video cameras with the standard definition mode are connected to media server, which is equipped with real-time H.264 encoding boards. Media server captures the video of each camera. At the same time, in MVV-SA, the audio is captured by microphone equipped with each camera. In MVV-A, it is captured by a microphone. Media server sends the audio and video of a viewpoint to media receiver by using UDP packets. Media receiver receives these packets and outputs the audio and video decoded from them. Media receiver can choose one viewpoint from the four cameras by sending a request with SUBSCRIBE method of SIP (session initiation protocol).

The NIST Net software is a network emulator. We use it for giving additional delay in the network.

Load server generates UDP packets including 1472 bytes payload with exponentially distributed interval and sends them to corresponding load receiver.

**Figure 4:**
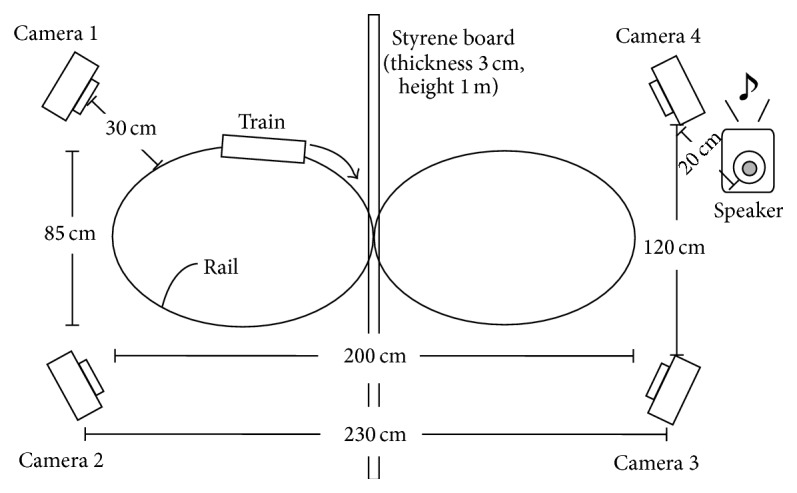
Camera arrangement.

The camera arrangement is shown in Figure 4. In the subjective experiment, an assessor watches a toy train which runs on plastic rails with changing the viewpoint. Each experimental run starts with the video and audio of Camera 1.

A speaker is put on 20 cm away from Camera 4, and music is played from there, because we assume background music. Moreover, as shown in Figure 4, a partition of styrene foam is put on the middle of the field so the user cannot see the other side of the field. When the user watches the video on Cameras 1 or 2, he/she cannot see the right side in Figure 4 and vice versa. We assume content with a large field like in sport broadcasting.

### 3.2. Audio and Video Specification and Experimental Parameter

The specifications of audio and video are shown in Table 1. We refer to the transmission unit at the application level as an MU (media unit). A video MU is a video frame and an audio MU is 960 audio samples. Each MU is transmitted as a UDP packet. We employ frame skipping as the output method of video. That is, when some packets consisting of an MU are lost, output of the MU is skipped.

In this paper, playout buffering control is used for absorbing delay jitter in media receiver. In the MVV-A system, playout buffering control brings tradeoff between the viewpoint change response and output quality [7]. In order to investigate the optimal playout buffering time, we employ five values of the playout buffering time in the experiment.

We assume two values of the average amount of UDP load traffic: 3.5 Mbps and 4.8 Mbps. They are selected on the basis of [10], which reveals that the amount of daytime traffic is about 70% of that of nighttime traffic. We have realized a situation in which congestion sometimes occurs between the two routers in Figure 3 on the nighttime traffic condition; as considering this situation, we set the average amount to 4.8 Mbps. The amount of daytime traffic is selected to be 3.5 Mbps, which is about 70% of 4.8 Mbps.

The NIST Net delays packets going through Router 1 and Router 2. By adding this delay, we can examine the effect of network delay on the QoE in MVV-SA and MVV-A. The delay was set to one of the two constant values: 0 ms and 150 ms. We assume the value of 0 ms as communications delay inside a city and the values of 150 ms as the latency of international communications from Japan to east-side of USA and UK. These values have been selected from [11], where the one-way delay from Japan to USA has a wide distribution from 60 ms to 150 ms; as for the delay from Japan to UK, the peak of the distribution is around 150 ms.

In this paper, we compare MVV-SA with MVV-A. In MVV-SA, the user can select audio and video according to the viewpoint. On the other hand, MVV-A disables audio selection; the user listens to audio captured on Camera 1.

### 3.3. Assessment Method

In the assessment, we ask the users to watch the running toy train. In this paper, we employ two kinds of average load, five kinds of playout buffering time, two kinds of additional delay, and MVV-SA or MVV-A. In total, we consider 40 stimuli obtained by these combinations. Before the experiment, assessors have confirmed a workflow of the task through practices. The total assessment time for an assessor is about 40 minutes including the practices and experimental runs. We employed 20 male students in their twenties as assessors.

In the experiment, we perform multidimensional QoE assessment with the SD (*semantic differential*) method [9]; it is a technique for evaluating an object from many aspects by means of many pairs of polar terms. The pairs of polar terms in the subjective experiment are shown in Table 2. The pairs are classified into six categories; there are three pairs for video, four pairs for audio, three pairs for psychology, a pair for response, a pair for synchronization, and a pair for overall satisfaction. Abbreviated names from v1 to o1 are attached to the pairs of polar terms.

Here, the pairs v2 and a3 (powerful–poor) are employed for asking dynamism of the video and audio, respectively. The pair p1 (free–restricted) is used for inquiring restriction in using the system, the pair p2 (simple–difficult) is for the assessment of complicatedness of the system, and the pair p3 (powerful–well-behaved) is for evaluating perceived atmosphere through the system.

Note that the experiment was performed with the Japanese language. This paper has translated the used Japanese terms into English from literature on subjective assessment and opinions of English speakers for better translation. However, the meanings of adjectives or verbs written in English here may slightly differ from those of Japanese ones because each language has its original background and culture.

For each criterion, a subjective score is measured by the* rating scale method* [12]. In the method, an assessor classifies the stimuli into a certain number of categories; here, each criterion is evaluated to be one of five grades. The best grade (score 5) represents the positive adjective (the left-hand side one in each pair), while the worst grade (score 1) means the negative adjective. The middle grade (score 3) is neutral.

The numbers assigned to the categories only have a greater-than-less-than relation between them; that is, the assigned number is nothing but an ordinal scale. When we assess the subjectivity quantitatively, it is desirable to use at least an interval scale. In order to obtain an interval scale from the result of the rating scale method, we first measure the frequency of each category with which the stimulus is placed in the category. With the law of categorical judgment [12], we can translate the frequency obtained by the rating scale method into an interval scale. Since the law of categorical judgment is a suite of assumptions, we must test goodness of fit between the obtained interval scale and the measurement result. Mosteller [13] proposed a method of testing the goodness of fit for a scale calculated with Thurstone's law of comparative judgment [12], which is one of psychometric methods. The method can be applied to a scale obtained by the law of categorical judgment. This paper uses Mosteller's method to test the goodness of fit. Once the goodness of fit has been confirmed, we refer to the interval scale as the psychological scale; it is a QoE metric.

## 4. Experimental Result

### 4.1. Application-Level QoS Parameters

In this subsection, we show the assessment results of application-level QoS parameters which represent the temporal quality of audio and video. We employ the viewpoint change delay and MU loss ratio as the application-level QoS parameters. The viewpoint change delay is defined as the time in seconds from the moment the destination sends a request for viewpoint change until the instant a new viewpoint is output at the destination. The MU loss ratio is defined as the ratio of the number of MUs not output to the total number of MUs transmitted.

**Figure 5:**
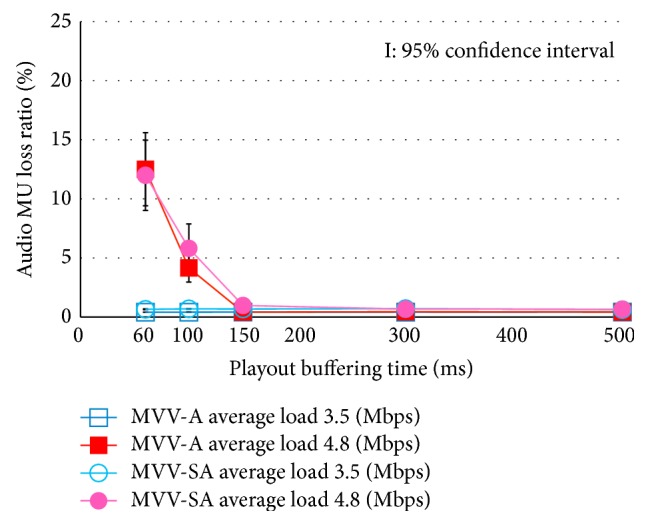
Audio MU loss ratio.

**Figure 6:**
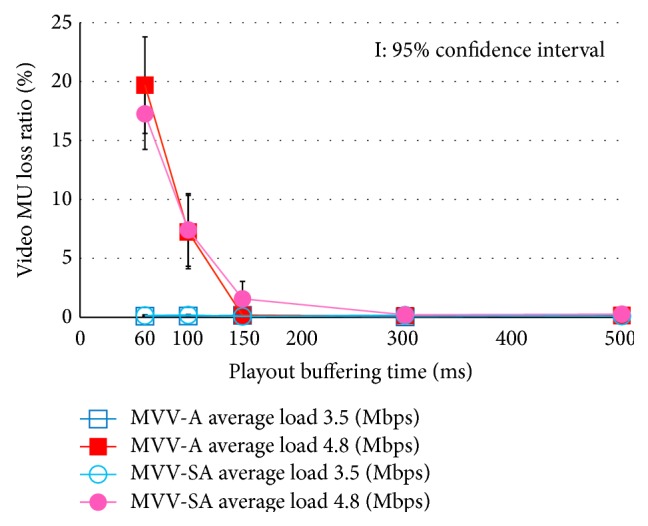
Video MU loss ratio.

Figure 5 depicts the MU loss ratio of audio, and Figure 6 represents that of video. The abscissa of these figures is the playout buffering time. In the following discussion, we regard the average load traffic 3.5 Mbps and 4.8 Mbps as the light load traffic and the heavy load traffic, respectively.

In Figures 5 and 6, we notice that the MU loss ratio is almost 0 under the lightly loaded traffic condition. On the other hand, under the heavy load condition with shorter playout buffering time than 150 ms, the MU loss occurs. This is because the delay jitter cannot be absorbed in the short buffering time. Under the condition, the MU loss ratio of video is larger than that of audio, because the MU size of video is larger than that of audio.

**Figure 7:**
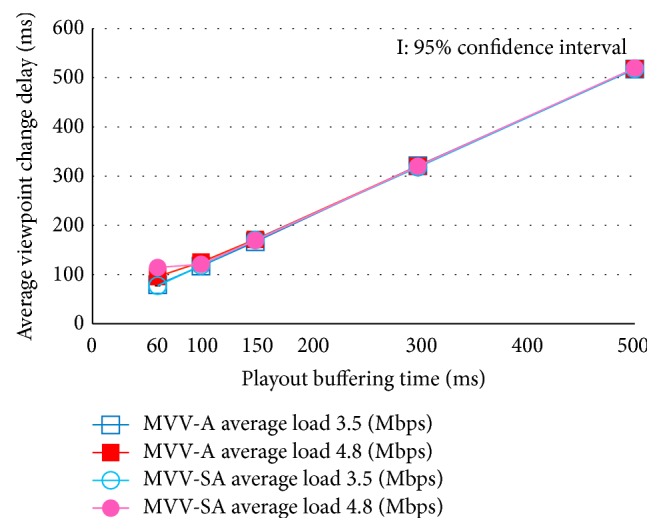
Average viewpoint change delay: additional delay 0 ms.

**Figure 8:**
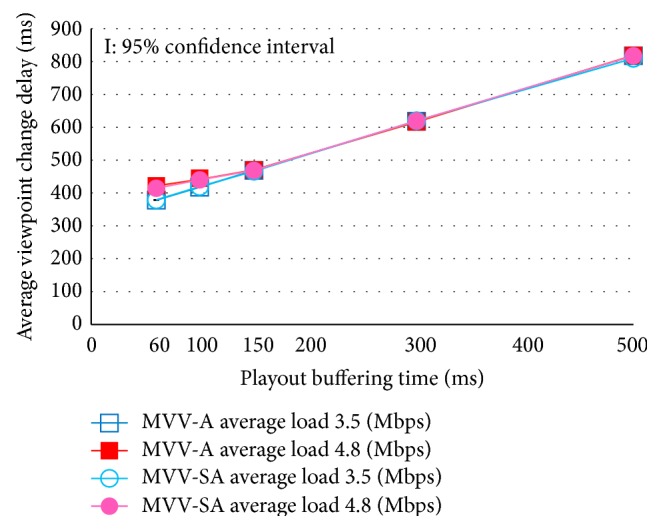
Average viewpoint change delay: additional delay 150 ms.

Figure 7 shows the average viewpoint change delay with the additional delay 0 ms.  Figure 8 depicts the average viewpoint change delay with the additional delay 150 ms.

We find in these figures that, in the case of playout buffering time 60 ms and 100 ms with load traffic 4.8 Mbps, the average viewpoint change delay is a little larger than the cumulated time of round trip time and the playout buffering time. This is because, with short buffering time and large delay jitter, a few MUs of the new viewpoint can be discarded owing to their delayed arrival. When this happens, the user may feel slow viewpoint changes because the video freezes until the new viewpoint is displayed.

### 4.2. Psychological Scale

In this paper, we obtained the psychological scale for each criterion (adjective pair) by the processes explained in Section 3.3, that is, the law of categorical judgment and the Mosteller's test. When we found that the hypothesis that the observed value equals the calculated one can be rejected with significance level of 0.05 by the Mosteller's test; we removed the stimuli which have large errors until the hypothesis cannot be rejected. For example, we removed three stimuli for “o1: excellent–bad”. This means that, except for the removed three stimuli, it is proper to consider the obtained interval scale as the psychological scale.

Since we can select an arbitrary origin in an interval scale, for each criterion, we set the minimum value of the psychological scale to unity.

**Figure 9:**
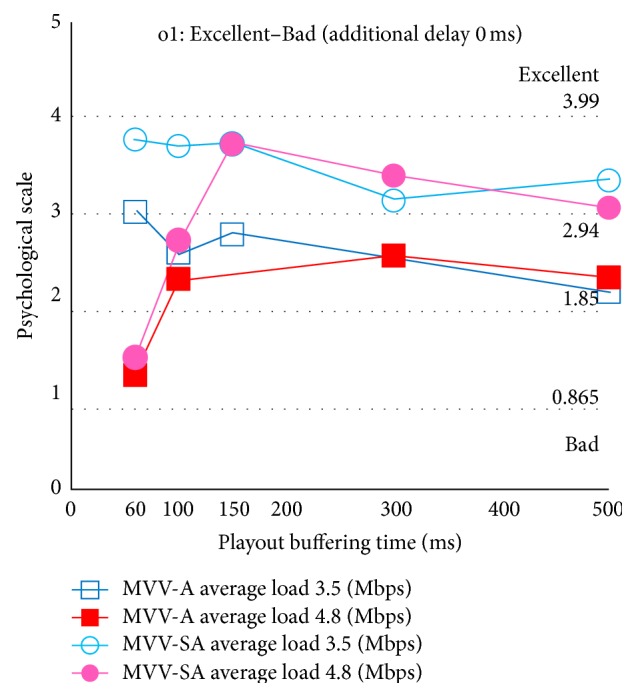
Overall satisfaction: additional delay 0 ms.

**Figure 10:**
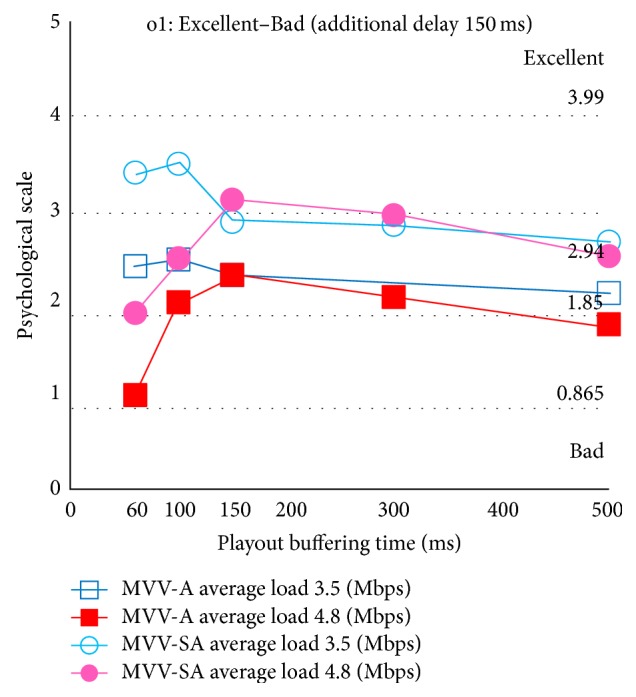
Overall satisfaction: additional delay 150 ms.

Figures 9 and 10 show the psychological scale for overall satisfaction with additional delay 0 ms and 150 ms, respectively. The abscissa is the playout buffering time, and the ordinate is the psychological scale. The dotted lines parallel to the abscissa depict boundaries of categories. In these figures, the removed stimuli through the Mosteller's test are not shown.

At first, we focus on the effect of selectable audio. In Figures 9 and 10, we notice that, for all the experimental conditions considered here, MVV-SA has higher psychological scale values than MVV-A. MVV-SA tends to exist in higher category than MVV-A; it is a quantitative advantage of MVV-SA. Thus, the selectable audio can enhance QoE in multiview video and audio transmission.

Next, we consider the effect of load traffic. In the case of light load traffic (i.e., average load 3.5 Mbps), as the playout buffering time increases, the overall satisfaction decreases. This is because the response of viewpoint change becomes worsen as the playout buffering time increases.

On the other hand, in the case of heavy load traffic (i.e., average load 4.8 Mbps), for almost all the experimental conditions considered here, the psychological scale for playout buffering time 150 ms is the maximum. This is because in the case of playout buffering time 60 ms and 100 ms, the user perceives deterioration of the audiovisual quality by congestion. On the other hand, in playout buffering time 300 ms and 500 ms, although the quality of audiovisual does not deteriorate with enough buffering time, the user's overall satisfaction decreases owing to slow response.

Moreover, we focus on the effect of the additional delay. We notice that when we employ the same playout buffering time, the psychological scale for additional delay 0 ms is higher than that for additional delay 150 ms. This is because, in the case of additional delay 0 ms, the user feels that the viewpoint change is fast and then can be satisfied for the system.

From the above discussion, we conclude that the user can get higher satisfaction by means of the selectable audio in the MVV-SA system. Furthermore, we notice that the users are sensitive to the tradeoff relation between the audiovisual quality and the viewpoint change response.

### 4.3. Factor Analysis

From the evaluation results obtained by the subjective experiment, in order to investigate what factors consist of the users' QoE, we conduct the factor analysis. The evaluation results of the adjective pairs (Table 2) except for “o1: excellent–bad” are used for the analysis.

As a result of the analysis, we picked up three components which have eigenvalues larger than unity. In order to interpret factors easily, we adopted the varimax rotation method.  Table 3 shows the contribution rate of each factor after the rotation. We obtained that the value of the KMO (Kaiser-Meyer Olkin) measure of sampling adequacy is 0.838; it means that the factor analysis is appropriate.

The factor loadings shown in Table 4 express the correlation between the factor and the adjective pair after the rotation. We then interpret the meaning of each component factor from the table.

The first factor largely affects the following three adjective pairs: “a2: the audio is large–the audio is small,” “a3: the audio is powerful–the audio is poor,” and “p3: I feel powerful–I feel well-behaved.” Thus, we can consider the first factor is “audio presence.”

We notice that “v3: the video is sharp–the video is blurred,” “v1: the video is smooth–the video is rough,” and “v2: the video is powerful–the video is poor” have high factor loadings for the second factor. Hence, the second factor can be regarded as “video quality.”

As for the third factor, “r1: the viewpoint change response is fast–the viewpoint change response is slow” and “p2: I feel simple–I feel difficult” are the most heavily loaded pairs on the factor. From the pairs, we consider that the third factor is “ease of viewpoint change.”

We also conducted multiple regression analysis in order to show the relationship among the component factors and overall satisfaction, that is, “o1: excellent–bad.” We regard the three factors denoted by *S*_1_, *S*_2_, and *S*_3_, respectively, as the independent variables, and the overall satisfaction denoted by *U*_o1_ as the dependent variable. We then obtained the following equation; here, *R* ^*∗*2^ denotes the contribution rate adjusted for degrees of freedom:

(1) Uo1=2.743+0.461S1+0.310S2+0.363S3hhhhhhhhhhhhhhhhihhhhR∗2=0.961.

From the high *R* ^*∗*2^ value, we can consider that the three factors can calculate overall satisfaction with high accuracy. We also find that the first factor, that is, audio presence, has the largest coefficient among the three factors. Thus, in MVV-SA, the presence by means of selectable audio can affect QoE largely.

**Figure 11:**
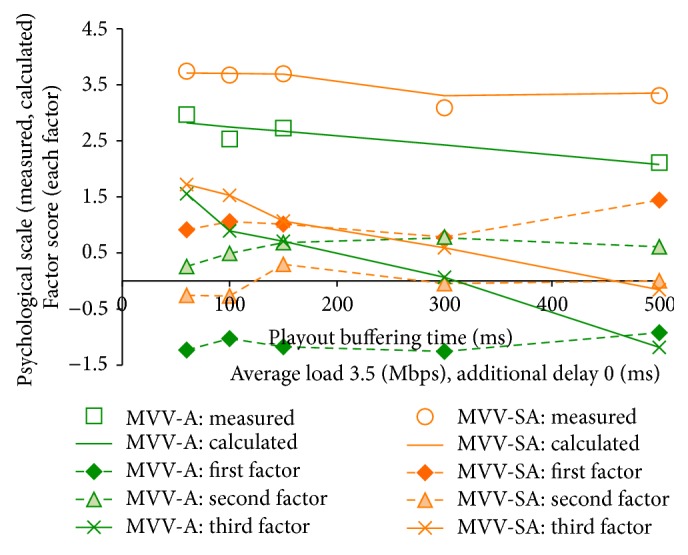
Psychological scale for overall satisfaction and factor scores.

Figure 11 plots the calculated values obtained by the equation along with the measured ones in MVV-A and those in MVV-SA with the average load 3.5 Mbps. The figure also shows the scores of each factor and does not depict the removed stimuli through the Mosteller's test. We notice in the figure that the psychological scale of overall satisfaction can be calculated with the three components.

In Figure 11, we find that the third factor degrades as the playout buffering time increases. As we discussed earlier, “r1: the viewpoint change response is fast–the viewpoint change response is slow” and “p2: I feel simple–I feel difficult” are correlated with the third factor largely. The two criteria degrade as the playout buffering time increases because the viewpoint change delay increases. Thus, the third factor also degrades according to the increment of the buffering time.

We also see in the figure that the first factor, that is, the audio presence, has positive scores in MVV-SA while the scores are negative in MVV-A. We then consider that the factor can show the advantage of MVV-SA appropriately.

## 5. Conclusions

In this paper, we compare MVV-SA, in which audio is selectable according to viewpoint change, with MVV-A, in which audio is unselectable, from a QoE point of view. As a result, we find that MVV-SA can acquire high users' satisfaction because the users can select audio with video. From the factor analysis, the factors which affect users' satisfaction are the audio presence, the video output quality, and the ease of viewpoint change.

As our future work, we will devise another function to enhance QoE of the MVV-SA system, for example, multiple screens. In addition, we need to evaluate QoE in other kinds of contents. We will also consider the tradeoff relationships between the cost of additional devices (microphones/cameras) and the enhancement of QoE.

## Figures and Tables

**Table 1:** Specifications of audio and video.

	Video	Audio
Coding method	H.264 (704 × 480)	Linear PCM
		(48 kHz, 16 bit)
Picture pattern	I	—
Average bit rate	4 [Mbps]	768 [kbps]
Average MU rate	30 [MU/s]	50 [MU/s]

**Table 2:** Pairs of polar terms.

Category	Pair of polar terms
Video	v1: the video is smooth–the video is rough
	v2: the video is powerful–the video is poor
	v3: the video is sharp–the video is blurred

Audio	a1: the audio is natural−the audio is artificial
	a2: the audio is large–the audio is small
	a3: the audio is powerful–the audio is poor
	a4: the audio is comfortable−the audio is jarring

Psychology	p1: I feel free–I feel restricted
	p2: I feel simple–I feel difficult
	p3: I feel powerful–I feel well-behaved

Response	r1: the viewpoint change response is fast−the viewpoint change response is slow

Synchronization	s1: the audio and video are in synchronization–the audio and video are out of synchronization

Overall satisfaction	o1: Excellent–Bad

**Table 3:** Contribution rate.

Factor	Eigenvalue	Contribution rate
1	4.663	38.856
2	3.944	32.870
3	2.560	21.333

**Table 4:** Factor loadings.

Adjective pair	Factor loadings		
	First	Second	Third
v1	0.190	0.904	0.261
v2	0.298	0.895	0.270
v3	0.173	0.912	0.204
a1	0.820	0.480	0.216
a2	0.958	0.082	0.073
a3	0.953	0.141	0.185
a4	0.754	0.519	0.255
p1	0.590	0.428	0.654
p2	0.188	0.429	0.850
p3	0.843	0.320	0.398
r1	0.191	0.169	0.884
s1	0.557	0.686	0.367
